# Authors' Reply

**DOI:** 10.1371/journal.pbio.0030270

**Published:** 2005-08-16

**Authors:** Mark Stoneking, Brigitte Pakendorf, Hiroki Oota

**Affiliations:** **1**Max Planck Institute for Evolutionary AnthropologyLeipzigGermany; **2**University of TokyoKashiwaJapan

Waters [1] makes a number of points concerning our article [2], which, in our view, require clarification. First, Waters states that classifying Southeast Asian highland groups as either strictly horticultural or strictly foraging is overly simplistic, as most groups practice horticulture supplemented with some degree of foraging. While we are sympathetic with the view that subsistence strategy is more complicated than a simple dichotomy (indeed, one of the main messages of our paper is that a strictly foraging group such as the Mlabri may have practiced horticulture in the past), we wish to emphasize that the Mlabri are, indeed, quite different from the other Southeast Asian highland groups in that they have never, in either their recorded or oral history, practiced horticulture. It is this distinction, coupled with their extreme paucity of genetic diversity, that sets them apart from other groups in the area.

Second, Waters suggests that our comparison of the Mlabri with hill tribes from a different geographic region (Chiang Rai and Mae Hong Son provinces of Thailand) leads to our conclusion that “the Mlabri were isolated from these groups,” and that had we examined neighboring groups of the Mlabri, we might have reached a different conclusion. These statements misrepresent our work; in particular, we found that the Mlabri were not genetically distinct from other hill tribes for which we had data, as the mtDNA sequence, Y-STR alleles, and autosomal STR alleles of the Mlabri are all found in other groups. Moreover, this sharing pattern is in stark contrast to African foraging groups, such as the !Kung and Pygmies, who are genetically distinct from their horticultural neighbors. It is precisely this sharing of alleles between the Mlabri and other groups that is the basis for our suggestion that the Mlabri may have reverted to their current exclusively foraging lifestyle from a previous horticultural lifestyle, rather than having always been foragers.

Finally, Waters states that we claimed that our data “solidly” support the scenario of an extreme founder event from a horticultural group, followed by reversion to a foraging lifestyle, for the origin of the Mlabri. This is not true; we were careful to state that our data only suggest such a scenario. We agree with Waters that genetic analysis of neighboring groups of the Mlabri (in particular, the Tin Prai) would be useful to further evaluate the scenario we proposed for the origin of the Mlabri. And we clearly agree with Water's concluding statement concerning the importance of interactions between horticultural and foraging groups, as we make exactly that point in the penultimate sentence of our paper.
